# Gut microbiome differences in individuals with PTSD compared to trauma-exposed controls: a systematic review

**DOI:** 10.3389/fnins.2025.1540180

**Published:** 2025-02-24

**Authors:** Chantelle Winder, Ami Lodhia, Melissa Basso, Kathrin Cohen Kadosh

**Affiliations:** ^1^Social Brain and Development Lab, School of Psychology, University of Surrey, Guildford, United Kingdom; ^2^School of Psychology, The University of Sheffield, Sheffield, United Kingdom; ^3^School of Psychology, University of Surrey, Guildford, United Kingdom

**Keywords:** gut microbiome, PTSD, gut-brain axis, trauma, microbiome gut-brain axis, post traumatic stress disorder

## Abstract

Post-traumatic stress disorder (PTSD) is a common mental health disorder that can occur following exposure to a traumatic event, and is characterized by symptoms including intrusive memories, dissociation, and nightmares. PTSD poses significant suffering on the individual and can reduce quality of life substantially, however, its mechanisms are not fully understood. It has also been associated with gut abnormalities, such as with irritable bowel syndrome, indicating possible involvement of the gut microbiome and gut-brain axis. Whereas previous research has implicated the gut microbiome and microbiome gut-brain axis in various mental health disorders, the relationship between gut microbiome function and PTSD is unclear. Specifically, little is known about whether specific gut microbiome compositions can increase the risk of developing PTSD, or, vice versa, act as a protective factor for the individual. This systematic review aims to synthesize the literature looking at gut microbiome differences between individuals with PTSD and trauma-exposed controls (TEC) while exploring potential risk and resilience factors for development of the disorder. Three studies met the inclusion criteria, and results showed that all studies found differences in gut microbial taxa between PTSD and TEC groups yet varied in their taxonomic level and type. One study found a significant difference in diversity between groups, reporting lower diversity in PTSD, and two studies found certain taxa to be correlated with PTSD symptom severity: *Mitsuokella*, *Odoribacter*, *Catenibacterium* and *Olsenella* genera, and *Actinobacteria*, *Lentisphaerae* and *Verrucomicrobia* phyla. This review has important implications for potential novel treatments for PTSD which target the gut microbiome, for example psychobiotic dietary interventions such as prebiotics and probiotics. It also informs our understanding of potential risk and resilience factors for the disorder, such as certain gut microbiome compositions being potentially protective or increasing susceptibility. More research is needed, as currently sample sizes are small and confounding variables (e.g., diet) are not always controlled for.

**Systematic review registration:** The protocol was registered on PROSPERO, registration number: CRD42024530033.

## Introduction

Post-traumatic stress disorder (PTSD) is a mental health disorder which can occur in some individuals following a traumatic event, and includes symptoms such nightmares, flashbacks, intrusive memories, dissociation, avoidance behaviors, and emotional and/or physiological distress triggered by certain cues related to the traumatic event (American Psychiatric Association, 2013). PTSD is estimated to have a lifetime prevalence of 3.4–26.9%, with female sex, younger age, lower income and social disadvantage being risk factors (Schein et al., 2021; Koenen et al., 2017). PTSD can occur at any age, with symptoms lasting from months to many years. The disorder has significant impact on the individual, such as interpersonal problems, suicidal ideation, and frequent comorbidities with other mental and physical health conditions (American Psychiatric Association, 2013; Sareen, 2014). Recommended treatments include therapies such as trauma-focused cognitive behavioral therapy (CBT), which have been shown to be effective. However, not all individuals recover sufficiently, and dropout rates can be high (Watkins et al., 2018), highlighting a need for additional treatment options.

The gut microbiome refers to the collection of microbes (such as bacteria) living inside the human gut, and the gut-brain axis refers to the bidirectional communication between the gut and brain. Evidence suggests that the gut microbiome influences gut-brain communication, impacting brain and behavior, and could be modulated to treat stress-related disorders (Cryan and O’Mahony, 2011). The Vagus nerve (which has been proposed as a target for treating both psychiatric and gastrointestinal disorders) is one of the key ways in which the gut and brain are connected and communicate, with its activity being influenced by the gut microbiome, potentially impacting mood and anxiety (Breit et al., 2018). The microbiome gut-brain axis has been a rapidly growing area of research within psychology in recent years, with numerous mental health disorders being linked to the gut microbiome, for example depression, anxiety disorders and bipolar disorder (Alli et al., 2022; Simpson et al., 2021; Nguyen et al., 2021). This emerging evidence creates opportunities for novel interventions targeting the gut microbiome in the form of dietary interventions, probiotics (strains of live bacteria) and prebiotics (food compounds which aid the growth of beneficial gut bacteria). While still a new area of research, some studies have found efficacy for microbiome-targeting interventions in improving mental health outcomes. For example, Freijy et al. (2023)’s recent randomized controlled trial showed improvements in anxiety and stress after a high prebiotic diet and improvement in wellbeing after probiotic supplementation relative to placebo.

Research investigating the role of the gut microbiome in PTSD has only recently emerged. For example, He et al. (2024) found a potential causal association between the gut microbiome and PTSD, highlighting the possibility of a dysfunctional microbiome gut-brain axis associated with the disorder. Previously, PTSD has been linked to gastrointestinal disorders such as irritable bowel syndrome (Ng et al., 2018), further corroborating the existence of an association with the microbiome gut-brain axis. Current research has highlighted that gut dysbiosis (an imbalance or change in the gut microbiome composition) could predispose individuals to developing PTSD after a traumatic event, and advocates for the use of interventions targeting the gut microbiome (Leclercq et al., 2016). Similarly, in an interesting study using an animal model of PTSD, Tanelian et al. (2022) found that specific gut bacteria were associated with susceptibility and resilience to developing anxiety behaviors after stress exposure. Specifically, before stress exposure, ‘susceptible’ rats’ microbiome had a pro-inflammatory phenotype overall, and ‘resilient’ rats had a more anti-inflammatory phenotype. Although promising, this study used an animal model of PTSD, which has limited generalizability to human PTSD. However, humans with PTSD have been shown to have increased inflammation and proinflammatory biomarkers (Passos et al., 2015; Hori and Kim, 2019).

Considering this recent evidence, this systematic review aimed to synthesize human research investigating gut microbiome differences between individuals with PTSD and individuals who have experienced trauma but do not have PTSD (trauma-exposed controls). Particularly, the review aims to answer the following question: what are the gut microbiome differences between trauma exposed individuals with and without PTSD? Elucidating these differences could then provide insight into gut microbiome mediated mechanisms contributing to PTSD development after trauma exposure. The use of trauma-exposed controls as opposed to healthy controls is key in exploring potential resilience or susceptibility to the disorder, and understanding why, after trauma exposure, some people develop PTSD whilst others do not. Further, it could inform the development of novel interventions targeting the gut microbiome, such as dietary or psychobiotic interventions - a term first coined by Dinan et al. (2013) defined as ingested live organisms which benefit individuals with psychiatric illness.

## Methods

This systematic review was prepared in line with the Preferred Reporting Items for Systematic Reviews and Meta-Analyses (PRISMA) guidelines (Page et al., 2021). The protocol was registered on PROSPERO, registration number: CRD42024530033.

### Information sources and eligibility criteria

The following databases were searched: PsycINFO, Scopus, PubMED, Web of Science and PTSDpubs. Dates of search were April–June 2024.

Observational studies looking at gut microbiome differences between trauma-exposed individuals with and without PTSD were eligible. Criteria for inclusion were as follows: adult participants with PTSD and adult participants with trauma exposure but no PTSD (trauma-exposed controls: TECs), and gut microbiome measures (measures of composition and/or diversity) taken via stool samples (16S rRNA sequencing). Only papers in English were included. No criteria were applied to years of publication.

Intervention studies were not included as this review is purely interested in existing, observable differences between the two groups without the influence of interventions such as pro- or prebiotics. Animal studies were excluded. Unpublished studies and grey literature were also excluded.

### Outcomes

The primary outcome was differences in gut microbiome (composition and diversity) between PTSD vs. TECs. Secondary outcomes were associations between PTSD symptom severity and gut microbiome composition and/or diversity. Diversity is measured in terms of alpha (richness and evenness measures of the gut microbiome) and beta (similarity or differences in the gut microbiome of two communities) diversity (Willis, 2019; Kers and Saccenti, 2022). They are used as indicators of gut microbiome health whereby greater alpha diversity is thought to protect against pathogens and may benefit health (Spragge et al., 2023).

### Search strategy

The following is an example search strategy used, please see Supplementary material for full search strategies used for each database.

*((“gut microbiome” or “gut-microbiome” or “gut bacteria” or “gut microbiota”) and (“ptsd” or “post-traumatic-stress-disorder” or “post traumatic stress disorder” or “post-traumatic stress disorder” or “posttraumatic stress disorder” or “post-traumatic stress” or “post traumatic stress” or “posttraumatic stress”)).ab.*

### Selection process

**Figure 1:**
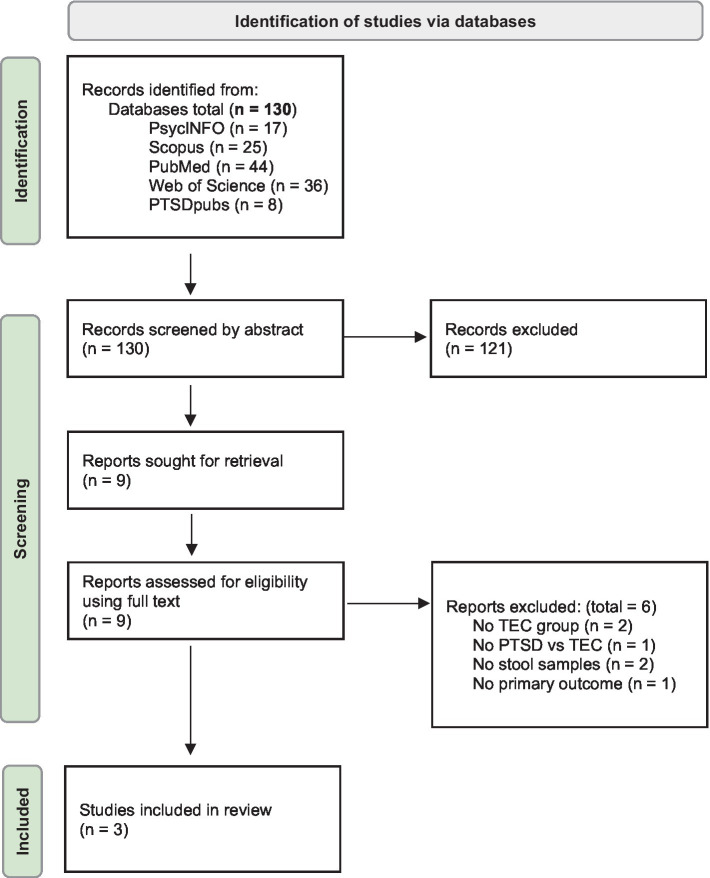
Flow diagram outlining the search process (Page et al., 2021).

Two independent reviewers (CW and AL) screened studies for eligibility based on the inclusion criteria. A third person (MB) resolved any disagreements. Studies were first screened based on title and abstract, then screened based on full text (see flow diagram in Figure 1 for details of the search process).

### Data collection process and data items

Data were extracted from the full text by two independent reviewers (CW and AL), with a third person (MB) resolving any disagreements.

Data extracted were: study information (authors, title, year, doi, sample size), participant characteristics (presence of adults with PTSD and trauma exposed controls, age, sex), study design (must be observational), measures (gut microbiome composition and/or diversity, measured using stool samples, PTSD diagnosis method) and study results (any microbiome differences between the two groups, both composition and diversity where applicable, and any secondary outcome results).

### Risk of bias assessment

ROBINS-E (Risk Of Bias In Non-randomized Studies of Exposures; Higgins et al., 2024) was used to assess risk of bias for each included study. This was done by two reviewers (CW and AL), with a third person resolving any disagreements (MB).

### Synthesis methods

Due to the small number of included studies and existing heterogeneity between results, a meta-analysis was not possible. A narrative synthesis was chosen to describe, discuss and compare findings from the included studies.

## Results

### Study selection

Of the 130 studies retrieved, three were eligible and included in the review (see Figure 1). One study met the inclusion criteria but was not included due to no full text being available (Hemmings et al., 2023).

### Study characteristics

Both Malan-Muller et al. (2022) and Hemmings et al. (2017) looked at gut microbiome differences between individuals with PTSD and trauma exposed controls. Bajaj et al. (2019) explored gut microbiome differences in veterans (all with active combat exposure) with and without PTSD (trauma exposed controls group), and all with cirrhosis.

Malan-Muller et al. (2022) did not find any differences for alpha diversity (measured using Simpson diversity index) or gut microbiome community composition variation (measured by Aitchison distance) between PTSD and TECs at genus or phylum levels. In a similar study, Hemmings et al. (2017) also found no differences between PTSD and TEC groups for alpha or beta diversity (measured using the Shannon index and ANOSIM). In contrast, Bajaj et al. (2019) found that diversity was lower in the PTSD group (measured using the Shannon diversity index), and PTSD was an independent predictor of lower diversity when controlling for cirrhosis severity.

Malan-Muller et al. (2022) found that a combination of *Mitsuokella*, *Odoribacter*, *Catenibacterium* and *Olsenella* genera were able to distinguish PTSD status with a 33.6% error rate, and the relative abundance of these was higher in the PTSD group than the TEC group. In terms of the secondary outcome, the relative abundance of these genera also correlated positively with CAPS-5 score (used to measure PTSD symptoms based on the DSM-5 criteria). Hemmings et al. (2017) found that *Actinobacteria*, *Lentisphaerae*, and *Verrucomicrobia* phyla could distinguish PTSD status, with a higher abundance being associated with lower probability of PTSD. For the secondary outcome, a decreased total abundance of these taxa was associated with higher CAPS-5 scores. In Bajaj et al. (2019)‘s study, the PTSD group had a lower relative abundance of potentially beneficial taxa from *Ruminococcaceae* and *Lachnospiraceae* families and increased relative abundance of pathobionts from *Enterococcus* and *Escherichia/Shigella*. A summary of the included study characteristics can be found in Table 1.

**Table 1:** Characteristics of each study included in the review.

Included study	Sample size	Sex (F%)	PTSD diagnosis method	Outcome measurement/method of analysis	Main results	
Malan-Muller et al. (2022)	PTSD (*n* = 79) TEC (*n* = 58)	PTSD = 79.74% TEC = 81.03%	CAPS-5/DSM-5	16S rRNA sequencing	Diversity: no difference between groups. Composition: *Mitsuokella*, *Odoribacter*, *Catenibacterium* and *Olsenella* genera higher in PTSD group.	Secondary outcome: abundance of these genera also correlated positively with CAPS-5 score.
Hemmings et al. (2017)	PTSD (*n* = 18) TEC (*n* = 12)	PTSD = 77.80% TEC = 58.30%	CAPS-5	16S rRNA sequencing	Diversity: no difference between groups. Composition: higher abundance *Actinobacteria*, *Lentisphaerae*, and *Verrucomicrobia* phyla associated with lower probability of PTSD.	Secondary outcome: decreased abundance of these associated with higher CAPS-5 scores.
Bajaj et al. (2019)	PTSD (*n* = 29) TEC (*n* = 64)	PTSD = 0% TEC = 0%	DSM-5	16S rRNA sequencing	Diversity: lower in PTSD. Composition: PTSD group had a lower abundance of potentially beneficial taxa from *Ruminococcaceae* and *Lachnospiraceae* families, and increased abundance of pathobionts from *Enterococcus* and *Escherichia/Shigella*.	

### Risk of bias assessment

ROBINS-E (Risk Of Bias In Non-randomized Studies of Exposures; Higgins et al., 2024) was used to assess the risk of bias in each study. It assesses risk of bias in seven domains: risk of bias due to confounding, from measurement of the exposure, in selection of participants, due to post-exposure interventions, due to missing data, arising from measurement of the outcome, and in selection of the reported result. Each domain is ranked as low risk, some concerns, high risk or very high risk, and an overall score is generated. See Table 2 for results of these assessments for each study (table generated using the Robvis tool; McGuinness and Higgins, 2020).

**Table 2:** Results of risk of bias assessments for each included study generated using the Robvis tool.

Study	D1	D2	D3	D4	D5	D6	D7	Overall
Malan-Muller et al. (2022)	HR	LR	SC	SC	LR	LR	LR	HR
Hemmings et al. (2017)	HR	LR	SC	SC	LR	SC	LR	HR
Bajaj et al. (2019)	HR	LR	SC	SC	LR	LR	LR	HR

D1: Domain 1 – Risk of bias due to confounding. D2: Domain 2 – Risk of bias arising from measurement of the exposure. D3: Domain 3 – Risk of bias in selection of participants into the study (or into the analysis). D4: Domain 4 – Risk of bias due to post-exposure interventions. D5: Domain 5 – Risk of bias due to missing data. D6: Domain 6 – Risk of bias arising from measurement of the outcome. D7: Domain 7 – Risk of bias in selection of the reported result. LR – low risk of bias. SC – some concerns. HR – high risk of bias. VHR – very high risk of bias.

## Discussion

The aim of this systematic review was to synthesise the current research looking at gut microbiome differences between individuals with PTSD and trauma exposed controls, which could help understand possible mechanisms behind the disorder as well as risk and resilience factors for development of the disorder.

In terms of differences in gut microbiome diversity, results were mixed: two of the studies reported no difference in diversity between PTSD and TEC groups, whilst one reported lower diversity in the PTSD group. As mentioned, gut microbiome diversity is used as an indicator of gut microbiome health, whereby increased diversity may protect against pathogens and benefit health (Spragge et al., 2023). Therefore, lower diversity in PTSD may negatively impact gut health and the gut-brain axis. More research is needed to explore this, although social stressors have been shown to reduce gut microbiome diversity in mice (Bailey et al., 2011).

All three studies found some differences in gut microbiome taxa between PTSD groups and TEC groups, but results varied in terms of taxonomic level and type: Malan-Muller et al. (2022) found *Mitsuokella*, *Odoribacter*, *Catenibacterium* and *Olsenella* genera to be higher in the PTSD group, Hemmings et al. (2017) found a higher abundance of *Actinobacteria*, *Lentisphaerae*, and *Verrucomicrobia* phyla to be associated with a lower probability of PTSD, and Bajaj et al. (2019) found the PTSD group had a lower abundance of potentially beneficial taxa from *Ruminococcaceae* and *Lachnospiraceae* families, and increased abundance of pathobionts from *Enterococcus* and *Escherichia/Shigella*. One explanation for the heterogeneity in these results could be the impact of confounding variables, which are not always controlled for. For example, Malan-Muller et al. (2022) do not control for diet, and Bajaj et al. (2019) do not control for presence of gastrointestinal disorders, both of which could have an impact on gut microbiome compositions.

In terms of the secondary outcome, two studies found certain taxa to be correlated with PTSD symptom severity: Malan-Muller et al. (2022) found that the abundance of *Mitsuokella*, *Odoribacter*, *Catenibacterium* and *Olsenella* genera correlated positively with CAPS-5 scores, and Hemmings et al. (2017) found that a decreased abundance of *Actinobacteria*, *Lentisphaerae*, and *Verrucomicrobia* was associated with higher CAPS-5 scores. Similarly, in another study, Zeamer et al. (2023) found that the gut microbiome accounted for 48% of the variance in PTSD raw scores, with the following found to be associated with PTSD symptom severity: abundance of *Firmicutes bacterium CAG:555*, *Bifidobacterium adolescentis*, and the proinflammatory *Streptococcus infantis*.

There are several limitations to be considered, notably the use of small sample sizes, particularly in Hemmings et al. (2017)‘s study. Additionally, the included studies are all cross-sectional in design, making it difficult to determine the role of the gut microbiome in PTSD over time, and to determine cause and effect. For example, it’s possible that having PTSD could lead to changes in the gut microbiome, rather than the other way around, which is supported by evidence that stressful events may induce gut dysbiosis (Gao et al., 2022). A longitudinal study by Feldman et al. (2022) - which followed trauma-exposed individuals for 15 years—found evidence to suggest a causative relationship between the gut microbiome and PTSD in terms of microbiome profiles for risk and resilience: gut microbiome composition and diversity could distinguish between individuals with PTSD and resilient individuals. Additionally, confounding variables affect research in this area, such as diet and presence of gastrointestinal disorders. Controlling for factors such as these would benefit future studies to reduce risk of bias and could be a potential cause of heterogeneity in results between studies.

This review has some limitations. Primarily, it includes only three studies, making it relatively small in scope. Nevertheless, the focus on studies that included TECs as a control group is particularly valuable for understanding why some individuals develop PTSD after experiencing trauma, as opposed to comparing individuals with PTSD to healthy controls. Specifically, using a TEC comparison group offers insights into the potential mechanisms underlying the development of the disorder following trauma, including risk and resilience factors and the influence of the gut microbiome. Furthermore, whilst studies using animal models of PTSD have found interesting gut microbiome differences (e.g., Zhou et al., 2020; Tanelian et al., 2022), more research in humans is needed.

The findings from this review align with previous literature suggesting a link between the gut microbiome and PTSD (e.g., He et al., 2024; Ke et al., 2023). However, more research is needed in this emerging area, particularly with the use of TECs as the key comparison group. This could have important implications for future novel interventions for PTSD, as well as potential protective strategies for those more vulnerable to developing the disorder, or those in high-risk situations for trauma exposure, e.g., individuals in the army or areas of conflict. In their review, Cowan et al. (2017) highlight the link between the gut microbiome and the amygdala, and advocate for interventions targeting the gut microbiome in amygdala related disorders, such as PTSD (Ousdal et al., 2020). Interventions targeting the microbiome, for example psychobiotic interventions such as probiotics, prebiotics and/or dietary interventions could provide an efficacious strategy (Leclercq et al., 2016). There is some evidence for psychobiotic interventions being effective in improving mental health symptoms (e.g., see Amirani et al., 2020), but to the authors’ knowledge no studies using psychobiotic interventions for PTSD have been conducted, except for a small pilot study by Brenner et al. (2020). This could be another beneficial and exciting area for future research.

Overall, this review provides important insights into gut microbiome differences between individuals with PTSD and TECs, improving our understanding of the development of the disorder, risk and resilience factors, and potential novel treatments targeting the gut microbiome. More research is needed in this new field, particularly controlling for confounding variables such as diet and gastrointestinal disorders, using larger sample sizes, and TECs as a control group.

## Data Availability

The data analyzed in this study is subject to the following licenses/restrictions: please contact the authors of the included studies for access to their datasets.
