# Supplementary material for: Gut microbiome differences in individuals with PTSD compared to trauma-exposed controls: a systematic review
Source: Front Neurosci. 2025 Feb 24;19:1540180. doi: 10.3389/fnins.2025.1540180 (PMC11891237; doi:10.3389/fnins.2025.1540180)
Supplement: Supplementary file 1 [file Data_Sheet_1.docx]

**Supplementary material**

*Full search strategies*

The following search strategies were used for each database:

PsycINFO:

*(("gut microbiome" or "gut-microbiome" or "gut bacteria" or "gut microbiota") and ("ptsd" or "post-traumatic-stress-disorder" or "post traumatic stress disorder" or "post-traumatic stress disorder" or "posttraumatic stress disorder" or "post-traumatic stress" or "post traumatic stress" or "posttraumatic stress")).ab.*

*limit 1 to human*

Scopus:

*( TITLE-ABS-KEY ( "gut and microbiome" OR "gut-microbiome" OR "gut and bacteria" OR "gut and microbiota" ) AND TITLE-ABS-KEY ( "ptsd" OR "post-traumatic-stress-disorder" OR "post and traumatic and stress and disorder" OR "post-traumatic and stress and disorder" OR "posttraumatic and stress and disorder" OR "post-traumatic and stress" OR "post and traumatic and stress" OR "posttraumatic and stress" ) ) AND ( LIMIT-TO ( EXACTKEYWORD , "Humans" ) )*

PubMED:

*(human microbiome[MeSH Terms] OR human microbiomes[MeSH Terms] OR microbiome, human[MeSH Terms] OR “gut microbiome”[Title/Abstract] OR “gut-microbiome”[Title/Abstract] OR "gut bacteria"[Title/Abstract] OR "gut microbiota"[Title/Abstract])) AND (ptsd[MeSH Terms] OR acute post traumatic stress disorder[MeSH Terms] OR chronic post traumatic stress disorder[MeSH Terms] OR delayed onset post traumatic stress disorder[MeSH Terms] OR post traumatic stress disorder[MeSH Terms] OR post traumatic stress disorders[MeSH Terms] OR "ptsd"[Title/Abstract] OR "post-traumatic-stress-disorder"[Title/Abstract] OR "post traumatic stress disorder"[Title/Abstract] OR "post-traumatic stress disorder"[Title/Abstract] OR "posttraumatic stress disorder"[Title/Abstract] OR "post-traumatic stress"[Title/Abstract] OR "post traumatic stress"[Title/Abstract] OR "posttraumatic stress"[Title/Abstract])*

*Filter applied: humans*

Wed of Science:

*(AB=((“gut microbiome” OR “gut-microbiome” OR “gut bacteria” OR “gut microbiota”) )) AND AB=((“ptsd” OR “post-traumatic-stress-disorder” OR “post traumatic stress disorder” OR “post-traumatic stress disorder” OR “posttraumatic stress disorder” OR “post-traumatic stress” OR “post traumatic stress” OR “posttraumatic stress”))*

*Filter applied: MeSH headings: humans*

PTSDpubs:

*abstract((“gut microbiome” OR “gut-microbiome” OR “gut bacteria” OR “gut microbiota”) ) AND abstract((“ptsd” OR “post-traumatic-stress-disorder” OR “post traumatic stress disorder” OR “post-traumatic stress disorder” OR “posttraumatic stress disorder” OR “post-traumatic stress” OR “post traumatic stress” OR “posttraumatic stress”))*
